# Hospital-Based Tap Water Iontophoresis for Primary Focal Hyperhidrosis: A Real-World Analysis of Treatment Adherence, Efficacy, and Relapse

**DOI:** 10.3390/jcm15020640

**Published:** 2026-01-13

**Authors:** Mizgin Gülmez, Berna Solak

**Affiliations:** 1 Department of Dermatology, Sakarya Training and Research Hospital, 54290 Sakarya, Türkiye; 2 Department of Dermatology, School of Medicine, Sakarya University, 54290 Sakarya, Türkiye

**Keywords:** primary focal hyperhidrosis, tap water iontophoresis, HDSS, treatment adherence, maintenance therapy

## Abstract

**Background:** Tap water iontophoresis (TWI) is a well-established second-line treatment for primary focal hyperhidrosis. While its efficacy is proven, data regarding the correlation between session frequency and clinical response, as well as long-term adherence in hospital-based settings, remain limited. **Objective:** We aimed to evaluate the efficacy and safety of hospital-based TWI and to analyze the relationship between the number of treatment sessions and clinical outcomes. **Methods:** This retrospective study included 92 patients with primary focal hyperhidrosis treated with TWI. Disease severity was assessed using the Hyperhidrosis Disease Severity Scale (HDSS). Clinical response was categorized as “Excellent” (≥2-point HDSS reduction), “Good” (1-point reduction), or “No Response.” Relapse rates and reasons for treatment discontinuation were analyzed over a 6-month follow-up period. **Results:** The overall objective response rate was 65.2% (46.7% Excellent, 18.5% Good). A significant positive correlation was found between the total number of treatment sessions and the degree of clinical response (r = 0.401, *p* < 0.001). Patients achieving an “Excellent” response completed a significantly higher median number of sessions compared to non-responders (*p* = 0.001). However, among responders, the relapse rate was 85% within six months. Logistical difficulties were the most common reason for treatment discontinuation (17.5%). No compensatory hyperhidrosis or severe adverse events were observed. **Conclusions:** Hospital-based TWI is a safe and highly effective induction therapy, with success rates closely linked to the number of completed sessions. However, the high relapse rate and logistical barriers to adherence suggest that hospital-based protocols should serve primarily as a bridge to home-based maintenance therapy to ensure sustained long-term remission.

## 1. Introduction

Primary focal hyperhidrosis (PFH) is a chronic autonomic disorder characterized by excessive sweating beyond physiological needs, significantly impairing patients’ quality of life, social interactions, and occupational productivity [1,2]. Although various therapeutic modalities exist, managing PFH remains challenging due to the limitations of current first-line and invasive options. Topical aluminum chloride, often the initial treatment choice, frequently fails in palmoplantar cases due to poor penetration and skin irritation, leading to high discontinuation rates [2,3]. While botulinum toxin injections offer efficacy, their widespread use is hindered by high costs, the pain associated with palmoplantar injections, and the temporary nature of the relief [4]. Furthermore, surgical interventions such as endoscopic thoracic sympathectomy, though providing permanent solutions, carry risks of serious complications, including compensatory hyperhidrosis, which can be more distressing than the original condition [5].

The prevalence of primary focal hyperhidrosis is estimated to affect approximately 4.8% of the population, imposing a substantial burden on quality of life [6]. While tap water iontophoresis (TWI) is primarily indicated for palmoplantar hyperhidrosis typically after the failure of topical antiperspirants, its exact mechanism of action remains not fully elucidated. Proposed mechanisms include the obstruction of sweat ducts due to hyperkeratinization or a functional disturbance of the sympathetic nerve transmission caused by the electrical gradient [7,8]. Various modalities exist, including tap water alone or the addition of anticholinergics like glycopyrronium bromide to enhance efficacy; however, tap water remains the most accessible first-line option in many hospital-based settings [7].

Given these challenges, Tap Water Iontophoresis (TWI) has long been recognized as a safe and effective non-invasive alternative, particularly for patients refractory to topical agents [2,4,9]. Despite its established role in treatment algorithms, there is a paucity of “real-world” data specifically evaluating the correlation between treatment adherence, defined by the total number of sessions, and the degree of clinical response in hospital-based settings.

In this retrospective study conducted at a tertiary care center, we aimed to evaluate the efficacy and safety of hospital-based TWI in a diverse cohort of patients with PFH. Specifically, we analyzed the relationship between the number of treatment sessions attended and clinical response rates, as well as the patterns of relapse, to better understand the impact of treatment adherence on therapeutic outcomes in a real-world clinical setting.

## 2. Methods

***Study Design and Population:*** This retrospective study was conducted by reviewing the medical records of consecutive patients diagnosed with primary focal hyperhidrosis who were treated with tap water iontophoresis (TWI) at our dermatology outpatient clinic between January 2018 and January 2023. Ethical approval for this study was obtained from the Non-Interventional Ethics Committee of Sakarya University Faculty of Medicine (Approval Number: E-71522473-050.04-372973-183). The study protocol adhered to the ethical principles of the Declaration of Helsinki.

Patients with complete medical records and a follow-up period of at least 6 months were included in the study. Exclusion criteria included evidence of secondary hyperhidrosis (e.g., thyroid disorders, infectious diseases, or malignancy), presence of metallic implants or pacemakers, pregnancy, and active dermatological infections in the treatment area.

***Treatment Protocol****:* All patients underwent TWI treatment using a direct current device (SWI-STO 2, KaWe, Asperg, Germany) with tap water at our dermatology outpatient clinic between January 2018 and January 2023. The hands and/or feet were immersed in trays filled with tap water, and the current intensity was adjusted to the patient’s maximum tolerance level (typically 10–20 mA) without causing pain, as described in standard protocols. Treatment sessions lasted approximately 20 min. The frequency and total number of sessions were recorded for each patient.

***Clinical Assessment and Evaluation of Efficacy:*** Pertinent demographic and clinical data, including age, gender, symptom duration, affected anatomical sites (palmoplantar or axillary), and specific treatment parameters, were systematically extracted from patient records. To assess the subjective severity of the condition, the Hyperhidrosis Disease Severity Scale (HDSS) was utilized [2]. This validated, single-item questionnaire asks patients to rate the interference of sweating with their daily activities on a four-point scale. Specifically, a score of 1 indicates sweating that is never noticeable and non-interfering, while a score of 2 represents tolerable sweating that sometimes interferes with activities. Higher scores denote greater severity, with a score of 3 indicating barely tolerable sweating that frequently interferes, and a score of 4 representing intolerable sweating that always interferes with daily life.

The primary measure of therapeutic efficacy was the change in HDSS scores from baseline to the conclusion of the treatment protocol. In accordance with established literature, a one-point reduction in the HDSS score correlates with approximately a 50% reduction in sweat production, whereas a reduction in two or more points corresponds to an 80% or greater reduction. Consequently, treatment response was stratified into three categories: an “Excellent” response was defined as an improvement of ≥2 points in the HDSS score; a “Good” response was defined as a 1-point improvement; and patients showing no change in their HDSS score were classified as having “No Response” [10].

For long-term efficacy assessment, patients were contacted via telephone to determine the duration of “dryness” (remission period) and relapse status. Relapse was defined as the return of sweating to baseline levels or levels requiring re-initiation of treatment.

***Statistical Analysis***: Statistical analyses were performed using SPSS software version 22.0 (IBM Corp., Armonk, NY, USA). Categorical variables (e.g., gender, response rates) were presented as frequencies and percentages, while continuous variables (e.g., age, number of sessions) were expressed as mean ± standard deviation (SD) or median (min–max) where appropriate. The Chi-square test or Fisher’s exact test was used to compare categorical variables between groups (e.g., response vs. gender). The relationship between the number of sessions and therapeutic response was analyzed using correlation coefficients (Spearman’s rho). A *p*-value of <0.05 was considered statistically significant.

## 3. Results

A total of 92 patients with primary focal hyperhidrosis were included in the final analysis. The study population consisted of 60 females (65.2%) and 32 males (34.8%), with a mean age of 21.7 ± 7.9 years (range: 10–52 years). The most frequently affected anatomical site was the palmoplantar region (*n* = 64, 69.9%), followed by the combined palmoplantar and axillary regions (*n* = 21, 22.8%) and the isolated axillary region (*n* = 7, 7.6%).

***Treatment Efficacy and HDSS Scores***: Following the treatment protocol, 43 patients (46.7%) achieved an “Excellent” response (≥2-point reduction in HDSS), and 17 patients (18.5%) achieved a “Good” response (1-point reduction). Consequently, the overall objective response rate (Excellent + Good) was 65.2%. Thirty-two patients (34.8%) showed no improvement (“No Response”).

Baseline disease severity was high across the entire cohort; all included patients had a pretreatment HDSS score of 3 or 4, with no significant difference in baseline scores between the response groups (*p* = 0.553). Following treatment, HDSS scores significantly decreased in the responder groups compared to the non-responder group (*p* < 0.001), confirming the clinical benefit in patients who adhered to the protocol (Table 1).

**Figure 1:**
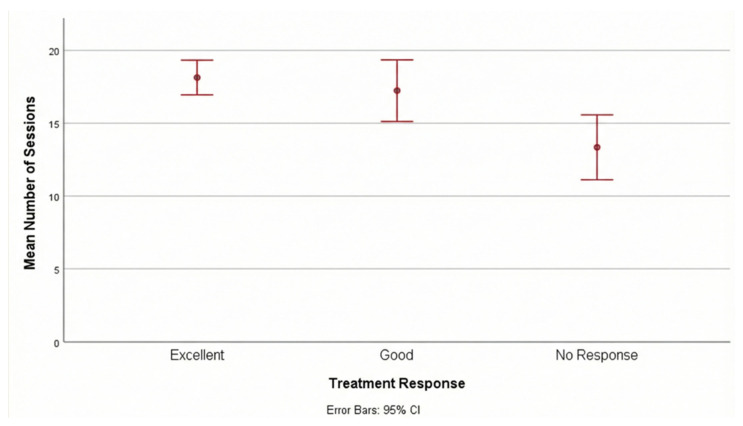
Mean number of sessions according to treatment response. The central dots represent the mean values, and the error bars represent the 95% Confidence Intervals (95% CI).

***Impact of Session Frequency on Outcome****:* A significant positive correlation was observed between the total number of treatment sessions and the degree of clinical response (r = 0.401, *p* < 0.001). Patients in the “Excellent” response group completed a significantly higher median number of sessions (median: 20, range: 6–20) compared to the “No Response” group (median: 13.5, range: 3–20) (*p* = 0.001). Figure 1 shows the mean number of sessions according to treatment response. This finding suggests that adherence to the treatment schedule and completion of a higher number of sessions are critical predictors of therapeutic success.

***Relapse and Follow-up:*** Among the 60 patients who responded to treatment (Excellent + Good), relapse was observed in 51 patients (85%) within the 6-month follow-up period. Of those who relapsed, 10 (19.6%) experienced a return of symptoms within the first month post-treatment. Notably, all 9 patients who did not experience relapse during the follow-up period were from the “Excellent” response group. Although the relapse rate was lower in the “Excellent” group compared to the “Good” group, this difference did not reach statistical significance (*p* = 0.050).

Subgroup analyses revealed no statistically significant differences in treatment response or relapse rates based on gender (*p* = 0.431) or affected anatomical site (*p* = 0.767) (Table 2 and Table 3).

***Safety:*** The treatment was generally well-tolerated with a favorable safety profile. Adverse events were rare; only one patient (1.1%) experienced mild, transient edema in the hands, which resolved spontaneously without necessitating discontinuation of therapy. No cases of compensatory hyperhidrosis, burns, or electrical shocks were reported. Regarding treatment discontinuation, patients in the ‘No Response’ group had a significantly lower median session count. The primary reasons for failing to complete the full 20-session protocol were logistical challenges (17.5%), including distance from the hospital and conflict with work/school hours, rather than intolerance to the procedure itself.

## 4. Discussion

In this real-world retrospective cohort of patients with primary focal hyperhidrosis, we demonstrate that hospital-based tap water iontophoresis (TWI) provides a clinically meaningful response in approximately two-thirds of patients, with an overall objective response rate of 65.2% and an excellent response in nearly half of the cohort. Beyond confirming the effectiveness and safety of TWI in routine clinical practice, the most important contribution of our study is the identification of treatment adherence, specifically the total number of completed sessions, as a key determinant of therapeutic success. We observed a moderate but highly significant positive correlation between session count and clinical response, with patients achieving an excellent response completing substantially more treatment sessions than non-responders. These findings highlight that the clinical effectiveness of TWI is not solely dependent on treatment initiation, but critically influenced by sustained engagement with the induction protocol. Our data further suggest that insufficient treatment exposure, rather than intrinsic treatment failure, may account for a substantial proportion of non-response observed in daily practice.

A pivotal finding of our study is the significant positive correlation between the number of treatment sessions attended and the degree of clinical response. Patients achieving an “Excellent” response completed a median of 20 sessions, whereas non-responders completed significantly fewer (median: 13.5). This observation supports the hypothesis that treatment adherence and a sufficient cumulative duration of therapy are critical for therapeutic success [7]. While some protocols suggest that clinical response can be achieved with fewer sessions [10,11], a recent study by Vural et al. [12] compared 10 versus 20 sessions of TWI. They reported that while both protocols resulted in similar gravimetric (objective) reductions, patients receiving 20 sessions reported significantly higher satisfaction (VAS) scores. This suggests that while 10 sessions may be effective in reducing sweat production, extending the regimen to 20 sessions might further enhance subjective symptom relief. This aligns with our observation that patients achieving an ‘Excellent’ HDSS response completed a significantly higher number of sessions.

Although our overall response rate of 65.2% confirms the efficacy of TWI, it is somewhat lower than the rates reported in some prospective clinical trials. For instance, Rahim et al. [2] reported a 97% response rate in a randomized controlled trial, and Kacar et al. [9] observed an 89.5% improvement in a pediatric cohort. Similarly, Dagash et al. [10] reported an 84% success rate in pediatric patients. The discrepancy between these high success rates and our findings may be attributed to the retrospective, “real-world” nature of our study compared to the strictly controlled environment of clinical trials. In our study, logistical barriers such as difficulty in visiting the hospital led to premature discontinuation in the non-responsive group, directly impacting the calculated efficacy. This mirrors the findings of Rajagopal et al. [4], where the initial response rate to TWI was 47%, potentially due to compliance issues in a non-invasive therapy arm.

Despite the initial efficacy, we observed a high relapse rate of 85% within the 6-month follow-up period, which highlights the temporary nature of TWI’s mechanism of action, presumed to be the functional blockade of sweat ducts or modulation of sympathetic transmission rather than permanent gland destruction [2,8]. The necessity for ongoing maintenance therapy is a well-documented challenge in hospital-based TWI protocols [10]. Gollins et al. [1] emphasized that while hospital-based treatment provides significant quality of life improvements, transitioning patients to home-based devices is crucial for long-term disease control. Our data, showing that 19.6% of patients relapsed within just one month, reinforces the recommendation that patients who respond well to the initial hospital-based course should be encouraged to acquire home devices to prevent logistical fatigue [1,10]. These findings align with Kim et al. [13], who similarly reported that maintenance is crucial given the variable relapse rates, confirming that hospital-based TWI should serve primarily as a bridge to long-term home maintenance.

Our analyses revealed that the therapeutic efficacy of TWI was independent of demographic variables and the anatomical site of hyperhidrosis (palmoplantar vs. axillary). No statistically significant differences were observed in treatment response, HDSS reduction, or relapse rates between genders or among affected sites. This suggests that TWI is a versatile option suitable for diverse populations [10,12]. In line with our findings, Vural et al. [12] reported no association between treatment response and gender, and Dagash et al. [10] demonstrated efficacy across both palmoplantar and axillary sites. Regarding safety, TWI was well-tolerated in our cohort with a favorable safety profile superior to other modalities; for instance, topical aluminum chloride frequently causes skin irritation [2,3], while botulinum toxin injections, though effective, are painful, expensive, and require repeated invasive procedures [4].

Our study has several limitations inherent to its retrospective design. First, the lack of a control group and the absence of gravimetric measurements limit the objectivity of the efficacy assessment, although HDSS is a validated patient-reported outcome. Second, there is a potential selection bias regarding the number of sessions; non-responders may have discontinued treatment earlier due to a lack of perceived benefit, which contributes to the lower session count in this group. Therefore, the correlation between session count and efficacy should be interpreted as a marker of adherence and ‘exposure to treatment’ rather than a comparison of fixed protocols. Finally, the sample size, while sufficient for main analyses, may lack power for smaller subgroup comparisons.

## 5. Conclusions

In conclusion, hospital-based tap water iontophoresis is a highly effective treatment for primary focal hyperhidrosis, with success rates closely linked to treatment adherence and session frequency. While it serves as an excellent induction therapy, the high relapse rates and logistical barriers necessitate a shift towards home-based maintenance strategies to ensure sustained remission. Clinicians should view hospital-based TWI as a bridge to patient education and home management rather than a standalone long-term solution.

## Figures and Tables

**Table 1:** Comparison of demographic and clinical characteristics, affected areas, and relapse status according to treatment response.

Variables	Excellent (*n* = 43)	Good (*n* = 17)	No Response (*n* = 32)	*p*-Value
Age (years), mean ± SD	21.1 ± 8.9	20.2 ± 4.7	23.2 ± 7.9	0.262
Gender, *n* (%)				
Female	31 (72.1%)	10 (58.8%)	19 (59.4%)	0.461
Male	12 (27.9%)	7 (41.2%)	13 (40.6%)	
Number of Sessions, median (min–max)	20 (6–20)	20 (6–20)	13.5 (3–20)	0.001 *
Affected Area, *n* (%)				
Palmoplantar	30 (69.8%)	14 (82.4%)	20 (62.5%)	0.733
Axillary	3 (7.0%)	1 (5.9%)	3 (9.4%)	
Palmoplantar + Axillary	10 (23.3%)	2 (11.8%)	9 (28.1%)	
Relapse within 6 months, *n* (%)	34 (79.1%)	17 (100%)	—	0.050
HDSS Score (Pre-treatment), median (min–max)	4 (3–4)	3 (3–4)	4 (3–4)	0.553
HDSS Score (Post-treatment), median (min–max)	2 (1–2)	2 (2–3)	4 (3–4)	<0.001 **
Change in HDSS Score, median (min–max)	2 (2–3)	1 (1–1)	0 (0–0)	<0.001 ^ɸ^

**Note:** Subgroup comparisons were performed using the Bonferroni correction. * Indicates a significant difference only between the “Excellent” and “No Response” groups. ** and ^ɸ^ Indicate significant differences among all three groups.

**Table 2:** Comparison of demographic characteristics, treatment response, and relapse rates according to the affected anatomical area.

Variables	Palmoplantar (*n* = 64)	Axillary (*n* = 7)	Palmoplantar + Axillary (*n* = 21)	*p*-Value
Age (years), mean ± SD	20.2 ± 6.4	31.1 ± 11.7	22.9 ± 8.8	0.061
Gender, *n* (%)				
Female	40 (62.5%)	4 (57.1%)	16 (76.2%)	0.503
Male	24 (37.5%)	3 (42.9%)	5 (23.8%)	
Number of Sessions, median (min–max)	20 (3–20)	20 (4–20)	20 (7–20)	0.981
Relapse within 6 months, *n* (%)	38 (86.4%)	3 (75.0%)	10 (83.3%)	0.685
Treatment Response, *n* (%)				
Excellent	30 (46.9%)	3 (42.9%)	10 (47.6%)	0.767
Good	14 (21.9%)	1 (14.3%)	2 (9.5%)	
No Response	20 (31.2%)	3 (42.9%)	9 (42.9%)	
HDSS Score (Pre-treatment), median (min–max)	4 (3–4)	4 (3–4)	4 (3–4)	0.490
HDSS Score (Post-treatment), median (min–max)	2 (1–4)	2 (1–4)	2 (1–4)	0.566
Change in HDSS Score, median (min–max)	1 (0–3)	1 (0–3)	1 (0–2)	0.867

**Table 3:** Comparison of demographic characteristics, treatment response, and relapse rates according to gender.

Variables	Female (*n* = 60)	Male (*n* = 32)	*p*-Value
Age (years), mean ± SD	22.1 ± 8.7	20.9 ± 6.3	0.452
Number of Sessions, median (min–max)	20 (3–20)	20 (3–20)	0.971
Affected Area, *n* (%)			
Palmoplantar	40 (66.7%)	24 (75.0%)	0.518
Axillary	4 (6.7%)	3 (9.4%)	
Palmoplantar + Axillary	16 (26.7%)	5 (15.6%)	
Relapse within 6 months, *n* (%)	37 (90.2%)	14 (73.7%)	0.126
Treatment Response, *n* (%)			
Excellent	31 (51.7%)	12 (37.5%)	0.431
Good	10 (16.7%)	7 (21.9%)	
No Response	19 (31.7%)	13 (40.6%)	
HDSS Score (Pre-treatment), median (min–max)	4 (3–4)	4 (3–4)	0.923
HDSS Score (Post-treatment), median (min–max)	2 (1–4)	3 (1–4)	0.395
Change in HDSS Score, median (min–max)	2 (0–3)	1 (0–3)	0.336

## Data Availability

The data that support the findings of this study are available from the corresponding author, upon reasonable request.
